# Integration of Machine Learning Algorithms and Discrete-Event Simulation for the Cost of Healthcare Resources

**DOI:** 10.3390/healthcare10101920

**Published:** 2022-09-30

**Authors:** Abdulkadir Atalan, Hasan Şahin, Yasemin Ayaz Atalan

**Affiliations:** 1 Faculty of Engineering, Gaziantep Islam Science and Technology University, Gaziantep 27260, Turkey; 2 Faculty of Engineering, Bursa Technical University, Bursa 16310, Turkey; 3 Faculty of Engineering, Yozgat Bozok University, Yozgat 66000, Turkey

**Keywords:** healthcare resources, *p_nt_* and *w_t_*, discrete-event simulation, machine learning, cost analysis

## Abstract

A healthcare resource allocation generally plays a vital role in the number of patients treated (*p_nt_*) and the patient waiting time (*w_t_*) in healthcare institutions. This study aimed to estimate *p_nt_* and *w_t_* as output variables by considering the number of healthcare resources employed and analyze the cost of health resources to the hospital depending on the cost coefficient (*δ*_i_) in an emergency department (ED). The integration of the discrete-event simulation (DES) model and machine learning (ML) algorithms, namely random forest (RF), gradient boosting (GB), and AdaBoost (AB), was used to calculate the estimation of the output variables depending on the *δ*_i_ of resources cost. The AB algorithm performed best in almost all scenarios based on the results of the analysis. According to the AB algorithm based on the *δ*_0.0_, *δ*_0.1_, *δ*_0.2_, and *δ*_0.3_, the accuracy data were calculated as 0.9838, 0.9843, 0.9838, and 0.9846 for *p_nt_*; 0.9514, 0.9517, 0.9514, and 0.9514 for *w_t_*, respectively in the training stage. The GB algorithm had the best performance value, except for the results of the *δ*_0.2_ (AB had a better accuracy at 0.8709 based on the value of *δ*_0.2_ for *p_nt_*) in the test stage. According to the AB algorithm based on the *δ*_0.0_, *δ*_0.1_, *δ*_0.2_, and *δ*_0.3_, the accuracy data were calculated as 0.7956, 0.9298, 0.8288, and 0.7394 for *p_nt_*; 0.8820, 0.8821, 0.8819, and 0.8818 for *w_t_* in the training phase, respectively. All scenarios created by the *δ*_i_ coefficient should be preferred for ED since the income provided by the *p_nt_* value to the hospital was more than the cost of healthcare resources. On the contrary, the *w_t_* estimation results of ML algorithms based on the *δ*_i_ coefficient differed. Although *w_t_* values in all ML algorithms with *δ*_0.0_ and *δ*_0.1_ coefficients reduced the cost of the hospital, *w_t_* values based on *δ*_0.2_ and *δ*_0.3_ increased the cost of the hospital.

## 1. Introduction

Resource allocation in healthcare services is a problematic issue in health institutions with a dynamic and complex structure. Hospital administrations have difficulties in terms of health management to carry out healthcare resource planning, especially in emergency departments (EDs) where there is no fixed number of patients or a certain patient arrival probability (in units that work without appointment) [1]. Due to the inability to correctly plan the healthcare resource allocation, many problems arise, such as patient waiting time ($w_{t}$), the number of patients treated ($p_{nt}$), and the cost of healthcare resources. In this study, a two-pronged solution was sought for the variation in the number of healthcare resources and the cost of the number of resources to the hospital to measure its effect on the ($p_{nt}$), and ($w_{t}$). In this study, this solution is provided by using the discrete-event simulation (DES) model and machine learning (ML) algorithms. A new method has emerged for estimation by using random forest (RF), gradient boosting (GB), and AdaBoost (AB) algorithms from ML algorithms, which extracts a wide variety of data attributes of parameters from the DES model.

By definition, DES is used to follow the activities by modeling the icons operating in a physical structure in the computer environment [2]. DES applications have a wide range of applications such as healthcare, energy, transportation, education, logistics, etc. Generally, the literature includes investigations of DES models for EDs. In this study, DES is modeled for the ED in a hospital, one of the most problematic units of the healthcare system because the events occurring in ED units are independent of one, and the next event is uncertain [3]. In addition, the fact that there are changes in the number of healthcare resources in EDs operating under the 24/7 working principle shows that it has a fluctuating structure.

The optimization of output parameters such as $w_{t}$, length of stay (LOS), performance, and $p_{nt}$ was carried out by considering all combinations with the experimental design of the number of healthcare resources employed in the ED using the DES model in research [3]. In another study $w_{t}$ by 40% and $p_{nt}$ by 28% were increased by creating the DES model in ED [4]. Cimellaro et al. succeeded in reducing the $w_{t}$ by 96% for patients with yellow codes and 75% for patients with green codes who applied to the ED unit by developing a Monte Carlo simulation model [5]. Researchers have run with simulation models not only to improve $p_{nt}$ or $w_{t}$ data, but also to improve healthcare resource performance. A study aimed to increase the efficiency of ED by developing a DES model and enabled the productivity of physicians and nurses to exceed 90% and 78%, respectively [6].

The DES model allows the results to be obtained quickly and inexpensively by making the changes that are thought to be made in the real system in the computer environment. The fact that the changes made in the healthcare system are not easy, and the results of the changes are obtained in the long-term have led to the use of DES. In a study, a new nurse-focused DES modeling technique was applied to predict nurse workload and quality of care. This study concluded that as NPR (the number of patients assigned to a nurse) increased, the quality of care deteriorated, and the nursing workload increased [7]. Another study was conducted with DES models to monitor the changes made in the behavior and performance of a set of operational policies for the efficient use and management of a scarce resource such as the intensive care unit (ICU) for the smooth operation of a hospital [8]. A cost–benefit analysis was carried out to measure the cost to the hospital of changes in the number of healthcare resources utilizing the DES model [9]. Baril et al. concluded that giving nurses more responsibility (bulk prescriptions, reviewing patients) significantly reduced the average time of LOS with less financial effort than adding extra doctors using the DES model for validating how nurses can contribute to reducing overcrowding in EDs [10].

ML algorithms are mostly used in medical subjects, although DES models are often preferred by researchers in solving healthcare resource problems, which are a significant problem in healthcare management. Ali et al. mentioned the importance of integrating DES into ML concepts and tools to improve the design and use of ML frameworks, arguing that this integration is an essential method of overcoming the difficulties of dynamic systems structures [11]. DES and ML integration methods should be used for EDs, which are the dynamic and complex units of the healthcare system. The integration of DES and ML algorithms in terms of healthcare management was provided and $p_{nt}$, $w_{t}$ and cost–benefit analyses were realized in this study.

ML, a subset of artificial intelligence (AI), has the ability to increase the accuracy of decisions through self-learning [12]. Today, ML algorithms are at the forefront of the methods used to draw conclusions and have the ability to predict data sets [13]. ML algorithms allow predictions for emerging situations using existing data, because ML algorithms are expressed as the science that gives the ability to learn automatically without programming exactly how to behave for each problem or system in the computer environment [14]. ML algorithms are also preferred in many areas such as business, healthcare, economy, production, and transportation. ML algorithms contain many algorithms that work according to different features.

Generally, ML models have been chosen to develop efficient decision support for health applications and to create an efficient decision support system [15]. In particular, these estimation approaches offer advantages in that they take into account nonlinear interactions of decision variables at a high rate among algorithms and obtain more stable estimation results [16]. We compared three different ML algorithms, including RF, GB, and AB, to find the most accurate model for the $p_{nt}$ and $w_{t}$ prediction data. The importance of the preferred prediction algorithms was also determined through the performance criteria of the ML models. In addition, these algorithms are preferred for accessing forecast data and providing forecasts, especially in structures with an uncertain (or stochastic) environment [17]. In the selected hospital unit in this study, these models were preferred in terms of fitting and evaluation since the characteristics of the time of arrival of the patients and the disease types were uncertain. We selected these models to represent a broad approach to the ML method along with the DES approach.

ML algorithms are the most important and trending method for innovation and predictive analytics in healthcare today to lead the digital healthcare transformation [18]. The authors of a study argued that ML is essential for making informed clinical decisions through insights from past data and for the core of evidence-based medicine [19]. Even if ML algorithms are used in many areas, positive results have been obtained by using ML algorithms in the field of healthcare, generally in medical subjects such as diagnosis, drug development, and treatment [20]. A study aimed to investigate using a support vector machine (SVM) from ML algorithms in predicting dementia and to validate its performance through statistical analysis [21]. Another study aimed to evaluate the completeness of reports of prognostic prediction models developed using ML methods in the field of oncology [22]. Ali and Aittokallio mentioned state-of-the-art ML methods for anticancer drug response modeling and prediction and better use of ML algorithms in high-dimensional multi-omics profiles with knowledge of targeted cancer pathways [23]. In another study, ML algorithms were developed to predict the response of cancer cell lines to drug therapy, quantified by IC50 (half maximal inhibitory concentration) values based on both the genomic characteristics of the cell lines and the chemical properties of the drugs under consideration [24]. ML algorithms have also been used on patients, healthcare resources, health life, patient safety, technologic innovation, health policies, etc., in the field of healthcare, apart from medical issues.

A study aimed to examine the usability of ML techniques to understand teamwork and behaviors related to healthcare management and patient safety and to contribute to the literature and research on collaboration in healthcare [25]. Another study discussed how developing an intelligent big data analytics platform based on ML and data integration principles is a new way to improve population healthcare management, value-based care, and upcoming challenges in healthcare [26]. Islam and Shamsuddin utilized the ML algorithms to develop a diagnostic system for patients with hypertension so that people can change their daily lifestyles to manage their condition [18]. Another study mentioned the application of ML to examine important issues such as fairness and transparency in healthcare modeling that directly impact operations and/or financial results in a hospital setting [27]. Gan reported on the overall value of prognostic reliability and principles of electronic healthcare management using ML techniques [28]. Another study described a population healthcare management tool based on ML algorithms, using administrative and socio-economic data for the early detection of high-risk patients [29].

Information on the objectives of some studies using the integration method of DES into ML algorithms in the healthcare field and the types of ML algorithms are given in Table 1.

ML algorithms should be used to measure the direct impact of healthcare resources on health service quality. One study used ML algorithms to understand the $w_{t}$ estimation behavior in two ED units by relating factors associated with $w_{t}$ estimation behavior and how it relates to patient flow modeling. At the same time, this study correlated the ML model with the DES technique, revealing that changing staffing patterns significantly reduced the overestimation of $w_{t}\;$ [30]. A researcher developed a ML-guided DES model to improve the processing of healthcare referrals. In this study, an estimation module for the application processing system was included to plan and prioritize referrals, reducing the average application generation latency by approximately 50% [31]. Another study used DES and ML algorithms to minimize $w_{t}$ in the emergency room and maximize the percentage of units’ participation to improve ED efficiency in a public hospital in Iran [32]. In one study, ML and DES models were integrated to analyze critical team capacity in the emergency coordination center (ECC) [34]. Different research aimed at a study that included DES and ML management to design health pathways and evaluate the return on investment of implementation [35]. Another study tried to solve the simulations of agile software processes for healthcare information systems development using ML methods [36]. Kim et al. used DES and ML algorithms to explore optimal thresholds to effectively triage COVID-19 patients in situations with limited data availability and minimize mortality while preserving healthcare system capacity [33]. The approaches proposed in the present article, the $p_{nt}$ and $w_{t}$ estimation data, the cost of a certain coefficient of healthcare resource personnel, and the cost to the hospital were examined in detail by attaching DES and ML algorithms.

The rest of the paper is as follows: Section 2 explains the data used, the integration of DES and ML algorithms, and how data can be integrated into the concepts and tools of this integration. Section 3 of the research comprises the results of the method applied in the study. Section 4 includes the necessity of the developed method in terms of applications and the discussion about the numerical results. The last part consists of some thoughts about the conclusion of the study.

## 2. Methodology

**Figure 1:**
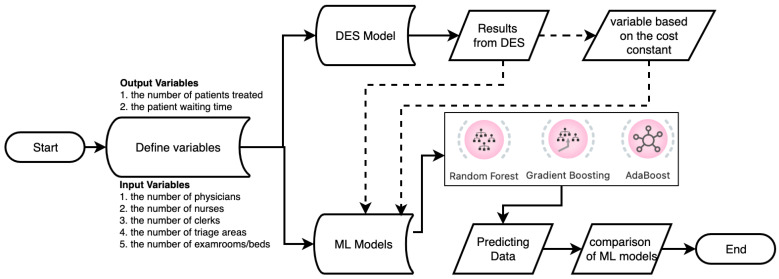
The flow chart of DES and ML models.

The methodology of this study consisted of two main stages. First, the data of the input/output parameters considered for the study were derived from the DES model. Data preprocessing was performed to avoid missing or erroneous data in the data set, and descriptive statistics results were calculated, because attention was paid to the regular and completeness of the data for the high performance of ML algorithms and the accuracy of the prediction data. Then, ML algorithms were used to obtain the estimated values of the output parameters by making changes to the input parameters. A total of 14,812 patients regarding to $w_{t}$ and 216 days for the data of $p_{nt}$ were used for each input and output parameter. The flow chart of the proposed methodology for health performance measurement modeling and mapping in the study is shown in Figure 1.

Figure 1 is a conceptual diagram of the workflow for an ED system that derives patient data for ML algorithms with the DES method. After preprocessing and filtering the patient data in the DES model, the data were used for prediction data in ML algorithms. It is crucial to run the DES model accurately to increase the accuracy of the results of the ML algorithms and to minimize the error rates. In the patient data used in both DES and ML algorithms, specific data such as gender, age, patient ID, address, and ethical committee approval were not used.

### 2.1. Data Collection and Its Characteristics

In this study, the treatment/examination flow of the patients was investigated by measuring the values of seven parameters. The primary output variables of the ML and DES models were the number of patients treated ($p_{nt}$) and the patient waiting time ($w_{t}$), which are continuous variables. $p_{nt}$ and $w_{t}$ are generally two important parameters for the management of hospitals. These parameters can be added to many parameters such as patient satisfaction, utilization rate of resource, length of stay so on. However, these two output variables are the basis of all indicators of healthcare quality. The first five variables of the seven parameters were used as the inputs to predict the $w_{t}$ and $p_{nt}$, the number of physicians ($p_{n}$), the number of nurses ($n_{n}$), the number of clerks ($c_{n}$), the number of exam rooms or beds ($b_{n}$), and the number of triage areas ($t_{n}$). Since the minimum and maximum values of $c_{n}$, and $t_{n}$ utilized in the ED were 1 and 2, two different integer values used for these input variables were binary. The computer-aided DES technique was used to calculate $p_{nt}$ and $w_{t}$ as output variables according to the values received by the sources of the ED unit of a hospital. There was no data property restriction for $w_{t}$ while actual and forecast data of $p_{nt}$ had integer properties. Table 2 shows the characteristics of the input and output variables in this study.

### 2.2. Discrete-Event Simulation

**Figure 2:**
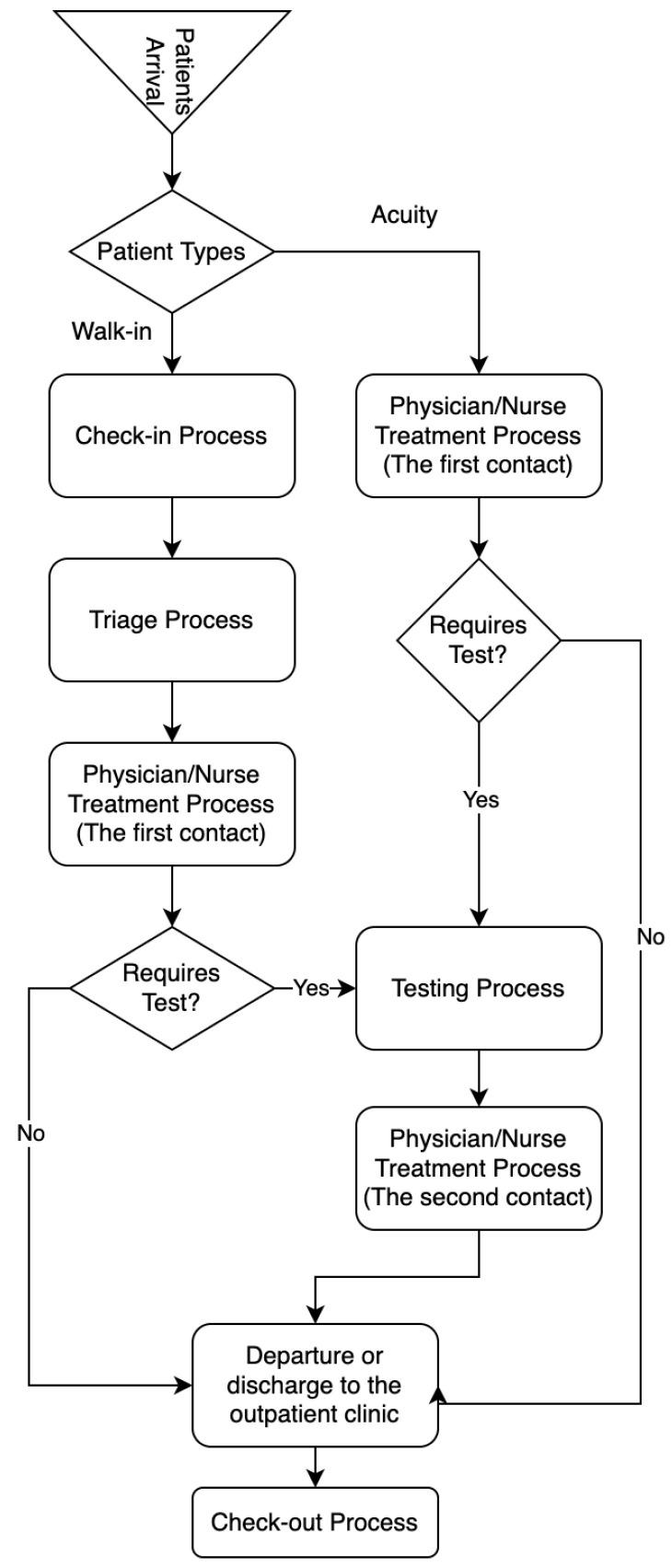
Patient flow chart created for the DES model.

The DES method is the most important tool for tracking objects in the system since mathematical models contribute to a limited extent in dynamic and complex systems. DES models are frequently preferred in many areas such as healthcare, energy, transportation and logistics, and production. Before the DES model is created, workflow diagrams that reveal the movements of objects in the system according to a certain rule should be created. In the DES model developed for the ED unit of a hospital, a workflow (patient flow: the processes that a patient has from the time of admission to the hospital until the time of leaving the hospital) diagram was created for the objects to move in a certain order. Figure 2 shows the patient flowchart considered for this study.

In this study, Flexsim HCE software, which works with 3D and pick-and-drop logic, was used to monitor the health resources employed in the ED unit. The main reason the DES technique was preferred in this study is that it is easy and cheap to observe the effects of changes made in a health unit on the health institution and to calculate the results such as cost, time, and efficiency. In this study, the values of $p_{nt}$ and $w_{t}$ were calculated as a result of the changes in the number of healthcare resources in the DES model. Information about the healthcare resource type and number used for the DES model is given in Table 3. Healthcare resources are defined in the literature as two types: human and location-based. In the DES model, physicians, nurses, and clerks were described as human-based; waiting areas, exam rooms or beds, and triage areas were expressed as location-based. In this research, a human-based resource was used for a unit in other locations except for the waiting room in the DES model.

In the DES model, the durations of the processes are calculated with the distribution. Since the arrival time and disease type of the patient arriving at the ED unit are unknown, it is inevitable to use the data as a distribution. The expert fit module processed the data collected for the DES model in the simulation program, and the distribution of the data sets was calculated.

**Figure 3:**
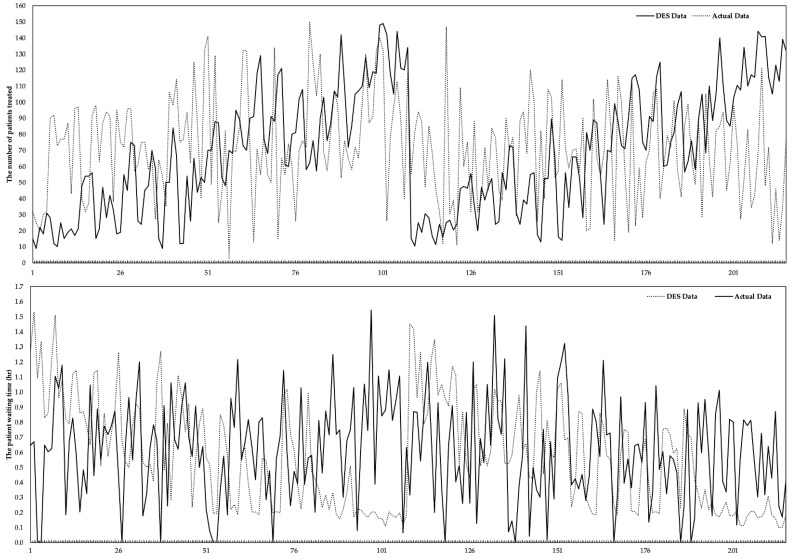
Verifying the output of the DES model.

The DES model was converted into basic three-dimensional animation using Flexsim HC simulation software for accuracy assurance in this study. The validation process of model-compatible operations was observed by running the developed model animation, monitoring the existence of all ED sources and strategies, and verifying the working system of the real ED system. The data in the real system were obtained by keeping the health resources in the ED unit constant. In contrast, the data in the DES model were obtained according to the scenarios depending on the number of healthcare resources. The results of the validation of the DES model (comparison with the actual $w_{t}$ and $p_{nt}$) are given in Figure 3. The difference between the actual and DES data of $p_{nt}$ was computed as 3.63%. The actual and DES data include the $w_{t}$ of the patients in the hospital’s ED until the end of the treatment/examination procedures and the end of the check-out process. The difference between both data sets was calculated as approximately 5.304%.

### 2.3. Machine Learning Models

**Figure 4:**
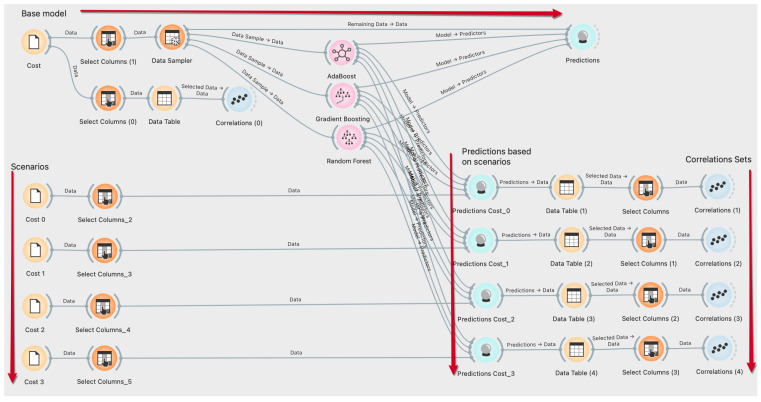
The flowchart of the ML algorithms of the study.

For this study, three ML algorithms, including RF, GB, and AB algorithms were utilized to predict the $p_{nt}$ and $w_{t}$ in different circumstances. Additionally, $p_{nt}$ and $w_{t}$ were estimated depending on the costs of healthcare resources using the classification features of the ML algorithms used in this study. The training and test data ratio was defined as 85%/15% for ML algorithms used to predict the data of the response variables. The cross-validation number of the ML algorithms was set as 10-fold layered for the prediction data. ML algorithms were utilized using orange software environment, open access, and phyton infrastructure, and prediction results were obtained. The algorithm created on the orange software of ML algorithms is shown in Figure 4. A detailed explanation of each algorithm is given in the following sections.

#### 2.3.1. Random Forest (RF)

The RF model is a regression tree model that uses bootstrapping aggregation and randomization of predictors to provide a highly accurate prediction result [37]. RF needs to use a set of decision trees to reduce the output variances of the trees [38]. The RF model shows high performance in models with a few random decision trees combined. The approximation values that add up by averaging the estimates and the number of input parameters are much higher [39]. An RF model was constructed by setting the randomly selected features and the number of trees to 5 and 10–2000, respectively.

#### 2.3.2. Gradient Boosting (GB)

GB model is a robust ML algorithm that shows high-performance success in practical applications in different fields such as energy, healthcare, economy, etc. This model can be highly customized according to some demands of the application, as well as being learned according to loss functions. For this reason, both loss function and basic learning models can be determined optionally in this model [40]. A single decision tree method may not be optimal for approximating smooth functions such as a linear trend since a single decision tree usually predicts the dependent/independent variable relationship with a constant value. For this reason, the GB model was preferred in this study to remove this limitation, increase the effect of the inputs on the output variable, and ultimately ensure the accuracy of the forecast data [41]. A GB model was created by hyper-features setting the randomly selected number of features: 100, the number of trees: 3, learning rates: 0.100, depth of individual trees: 3, not split subsets smaller than: 2, and the fraction of training instances: 1.00.

#### 2.3.3. AdaBoost (AB)

The AB algorithm was the first practical augmentation algorithm introduced by Freund and Schapire in 1996 [42]. The AB model is one of the most important and influential ML classification algorithms in reinforcement, computer vision, and pattern recognition because of its high generalization ability, fast performance, and low application complexity [43]. AB provides better accuracy than the decision tree when the number of class labels in the study dataset is considered to be two [44]. A GB model was created by hyper-features setting the number of estimators: 50, base estimator: tree, learning rates: 1.000, regression loss function: linear, and classification algorithm: SAMME.R (type of algorithm that uses probability estimates to update the additive model). Hastie et al. mathematically created SAMME and Algorithm 1, SAMME.R algorithms structures as follows [45]:
**Algorithm 1. SAMME Algorithm**  *Step 1. Initialize the observation weights*
$w_{i}=\frac{1}{n},\;i=1,2,\ldots ,\;n.$  *Step 2. For*
m=1 to M*:*  *Step 2.1. fit a classifier*
$T^{\left(m\right)}\left(x\right)$
*to the training data using weights*
$w_{i}$.  *Step 2.2. compute:*  $err^{\left(m\right)}=\sum_{i=1}^{n}w_{i}∥\left(c_{i}\neq T^{\left(m\right)}\left(x_{i}\right)\right)/\sum_{i=1}^{n}w_{i}$.  *Step 2.3. compute:*  $\alpha^{\left(m\right)}=log\frac{1-err^{\left(m\right)}}{err^{\left(m\right)}}+log\left(K-1\right)$.  *Step 2.4. set:*  $w_{i}\leftarrow w_{i}.exp(\alpha^{\left(m\right)}∥\left(c_{i}\neq T^{\left(m\right)}\left(x_{i}\right)\right),\;i=1,\;2,\;\ldots ,\;n$.  *Step 2.5. re-normalize*
$w_{i}$.  *Step 3. Output*  $C_{\left(x\right)}=arg\begin{array}{c} max\\ k \end{array}\sum_{m=1}^{M}(\alpha^{\left(m\right)}∥\left(T^{\left(m\right)}\left(x\right)=k\right)$.  ***SAMME.R algorithm:***  *Step 1. Initialize the observation weights*
$w_{i}=\frac{1}{n},\;i=1,2,\ldots ,\;n.$  *Step 2. For*
m=1 to M*:*  *Step 2.1. fit a classifier*
$T^{\left(m\right)}\left(x\right)$
  *to the training data using weights*
$w_{i}$.  *Step 2.2. obtain the weighted class probability estimates:*  $P_{k}^{\left(m\right)}\left(x\right)=Prob_{w}\left(c=k\setminus x\right),\;k=1,\;2,\;\ldots ,\;K$  *Step 2.3. set:*  $h_{k}^{\left(m\right)}(x)\leftarrow (K-1)\left(\log p_{k}^{m}(x)-1/K\sum_{k\prime }\log p_{k\prime }^{\left(m\right)}(x)\right),k=1,2,\ldots ,K$.  *Step 2.4. set:*  $w_{i}\leftarrow w_{i}.exp\;(-\frac{K-1}{K}y_{i}^{T}logp^{\left(m\right)}\left(x_{i}\right),\;i=1,\;2,\;\ldots ,\;n$.  *Step 2.5. re-normalize*
$w_{i}$.  *Step 3. Output*  $C_{\left(x\right)}=arg\begin{array}{c} max\\ k \end{array}\sum_{m=1}^{M}h_{k}^{\left(m\right)}\left(x\right)$.
where the input (predictor) variable is denoted as $x_{i}$ and the response (output) variable value is symbolized as $c_{i}$ in a finite set. These two variables are defined for the training dataset. $C_{\left(x\right)}$ is defined as the misclassification error rate. The best performance in this model is achieved with the lowest misclassification error rate. A weak multi-class classifier is represented as T(x). The $\alpha^{\left(m\right)}$ symbol ensures that the weights of the training samples are updated in the direction of the signs. The y denotes the two-class classification setting in y=(∥(c=1)−∥(c=2)) ∈ {−1, 1}. y was considered two-class since Friedman et al. established a relationship between the two-class AB algorithm and the exponential loss function [46]. An $h_{k}^{\left(m\right)}\left(x\right)$ is expressed as an improved estimate by minimizing the loss at each x.

### 2.4. Healthcare Resources Cost

Naturally, the resources employed in a business or institution have a cost to the business. Likewise, the resources utilized in health institutions should have a cost to the healthcare unit. The initial cost ($cost_{0.0}$) of healthcare resources to a healthcare unit is given in the following Equation (1):

(1) $$cost_{0.0}={\left[n_{r}\sum_{r}r\right]}_{s}\;r=\left\{(p_{n},n_{n},\;c_{n},\;b_{n},\;t_{n},\;\ldots \right\},\;n_{r}=\left\{1,\;2,\;3,\;\ldots ,\;n\right\}\;s=\left\{1,2,\ldots s\right\},\;\;$$

where the healthcare resource type is represented by r. The $n_{r}$ the symbol indicates the number of the same resource type working in a unit. The scenarios that occur as a result of the changes in the number of healthcare resources in a unit are expressed with s. In case of a one-unit increase in healthcare resource expenses, there must be a constant coefficient ($\delta_{i})$ in addition to Equation (1). So, Equation (2) is formed as follows:

(2) $$cost_{k}=\delta_{i}*{\left[n_{r}\sum_{r}r\right]}_{s}\;k=\left\{0.1,\;0.2,\;0.3,\;0.4,\;\ldots ,\;k\right\}\;\delta_{i}=\left\{ℛ>0=\{i\in ℛ|i>0\right\}$$

Equation (3) has been considered since the changes in healthcare resource costs will be based on previous costs,

(3) $$cost_{k}=\delta_{i}*{\left[n_{r}\sum_{r}r\right]}_{s}+cost_{k-1}$$

As a result of these developed equations, the effect on the estimated values of $p_{nt}$ and $w_{t}$ in an ED unit were determined. Although the constant coefficient is the most influential parameter on the output variables, different coefficients were applied for each healthcare resource. After the initial coefficient $\left(\delta_{0}\right)$ values were determined for each healthcare resource, a certain percentage increase was achieved for each $cost_{k}$. The coefficient values that were effective in the cost of healthcare resources to the ED unit are given in Table 4.

The results obtained in these equations (closed form), which belong to a dynamic and complex structure, can only be accepted as estimates. The main reason for this situation is that the values of the processes and patient admission rates of the ED unit contain statistical and stochastic expressions. As a result of these equations, the estimated values of $p_{nt}$ and $w_{t}$ were obtained by running scenarios with multipliers in ML algorithms. Equations (4) and (5) were constructed as follows to show the relationship between the cost of the current situation ($cost_{p_{nt}}^{c})$ to the hospital and the cost with a coefficient affecting the healthcare resource cost ($cost_{p_{nt}}^{s})$ for $p_{nt}$:

(4) $$cost_{p_{nt}}^{s}=\frac{\sum p_{nt}}{\left(p_{n}+n_{n}+c_{n}+b_{n}+t_{n}\right)}$$

(5) $$cost_{p_{nt}}^{s}=\frac{\sum p_{nt}}{\delta_{i}(p_{n}+n_{n}+c_{n}+b_{n}+t_{n})},\;i=\left\{0.0,\;0.1,\;0.2,\;0.3\right\}$$

Equations (6) and (7) were created as follows to express the cost of $w_{t}$ to the hospital according to the current situation ($cost_{w_{t}}^{c}$) and the ML algorithms of the cost depending on the $\delta_{i}$ coefficient of healthcare resources ($cost_{w_{t}}^{s}$):

(6) $$cost_{w_{t}}^{c}=\frac{\frac{\sum w_{t}}{p_{nt}}}{\left(p_{n}+n_{n}+c_{n}+b_{n}+t_{n}\right)}$$

(7) $$cost_{w_{t}}^{s}=\frac{\frac{\sum w_{t}}{p_{nt}}}{\delta_{i}(p_{n}+n_{n}+c_{n}+b_{n}+t_{n})},\;i=\left\{0.0,\;0.1,\;0.2,\;0.3\right\}$$

### 2.5. Model Metrics

The performance metrics are needed to measure the consistency and validity of the numerical results of ML algorithms. At the same time, these criteria are defined as statistical test criteria used to measure the effectiveness of ML algorithms during the testing and training phase [47]. The estimation data of ML algorithms are considered according to performance measures. The four performance measures are given below:

Root mean square error (RMSE):

(8) $$RMSE=\sqrt{\frac{\sum_{i=1}^{N}{\left(x_{a}-{\tilde{x}}_{e}\right)}^{2}}{N}}$$

Mean squared error (MSE):

(9) $$MSE=\frac{1}{N}\sum_{i=1}^{N}{\left(x_{a}-{\tilde{x}}_{e}\right)}^{2}$$

Mean Absolute Error (MAE):

(10) $$MAE=\frac{1}{N}\sum_{i=1}^{N}\left|x_{a}-{\tilde{x}}_{e}\right|$$

Correlations Coefficient (R):

(11) $$R^{2}-1=\sum_{i=1}^{N}{\left[\frac{x_{a}-{\tilde{x}}_{e}}{x_{a}-{\bar{x}}_{e}}\right]}^{2}$$

where the data size of the data set (observations) used for the testing and training phases is represented by *N*. The actual values are denoted by $x_{a}$. The symbols of ${\tilde{x}}_{e}$ and ${\bar{x}}_{e}$ represent the value of estimated and the average of actual values, respectively.

## 3. Results and Discussion

The RF, AB, and GB algorithms were utilized to predict the $p_{nt}$ and $w_{t}$ using the dataset of hospitalized patients in ED. A coefficient value affecting healthcare resource costs was used to derive the forecast data in ML algorithms. A total of 85% of the output data were used for training the models, and the remaining 15% of the data set was used to evaluate the estimation performance of the developed models. The comparison was performed between the initial data and the data obtained depending on the coefficient value.

### 3.1. Statistical Results

According to the ML model with the best output value performance, correlation results were evaluated with input variables. Table 5 shows the correlation values between the input and output variables as a result of the changes made in the coefficient affecting the healthcare resource cost. The correlation values have positive or negative values, indicating that the input variables have a positive or negative effect on the output variables. As a result of the changes in the amount of coefficient, it was observed that there was a fluctuating relationship between the output parameters and the input variables.

### 3.2. The Results of the Performance Evaluation

Each estimation algorithm tested had different values for each statistical measure during the evaluation phase. These values were interpreted for each ML algorithm. Accuracy (R^2^), MSE, RMSE, and MAE are the most emphasized statistical measures for comparison purposes in ML algorithms. The predictive models, RF, GB, and AB were evaluated using 10-fold layered cross-validation. The performance results of the preferred ML algorithms are shown in Table 6. Performance results of ML algorithms are handled differently according to two output variables. In addition, the performance values of each ML model were calculated for the training and testing sections.

For $p_{nt}$, the AB algorithm represented the best accuracy with 98.38% and 98.17% in the training and testing phases. RF and GB exposed 97.03% and 96.72% accuracy in the training phase and 95.39% and 95.02% in the testing phase, respectively. For $w_{t}$, the GB and RF algorithms had the lowest accuracy values of 92.05% and 92.75%. The AB algorithm reported a better accuracy of 94.92% in the training phase. While the AB and GB performance values were the same as in the training stage in the testing phase, it was observed that there was a slight change in the RF performance value. For two output variables, the AB algorithm had the lowest error values as well as having the best accuracy value in both phases.

The measurement performance values of the ML algorithms for $p_{nt}$ and $w_{t}$ based on $\delta_{i}$ are given in Table 7. The AB algorithm provided the best accuracy performance value for both output variables in the testing and training phases. According to the AB algorithm based on the coefficients $\delta_{0.0}$, $\delta_{0.1}$, $\delta_{0.2}$, and $\delta_{0.3}$, the accuracy data were calculated as 0.9838, 0.9843, 0.9838, and 0.9846 for $p_{nt}$; 0.9514, 0.9517, 0.9514, and 0.9514 for $w_{t}$ in the training phase, respectively. In the test phase, the GB algorithm had the best performance value, except for the results of the $\delta_{0.2}$ coefficient (the AB algorithm had the better accuracy at 0.8709 based on the value of $\delta_{0.2}$ for $p_{nt}$). According to the AB algorithm based on the coefficients $\delta_{0.0}$, $\delta_{0.1}$, $\delta_{0.2}$, and $\delta_{0.3}$, the accuracy data were calculated as 0.7956, 0.9298, 0.8288, and 0.7394 for $p_{nt}$; 0.8820, 0.8821, 0.8819, and 0.8818 for $w_{t}$ in the training phase, respectively.

Considering the statistical measurements with high percentages of accuracy and minimized margins of error in forecasting models, it emerged that the AB algorithm was more suitable than other algorithms. However, the estimation data of all three models for the two output variables are discussed in the follow-up of this section.

### 3.3. The Estimated Number of Patients Treated ($p_{nt})$

Balanced class weights were used for the prediction data of the RF, GB, and AB algorithms. The estimated data values obtained from these models were very close to each other. In addition to the health resource numbers of each algorithm, the estimated values were calculated according to the four scenarios created for health resource cost performance evaluations.

**Figure 5:**
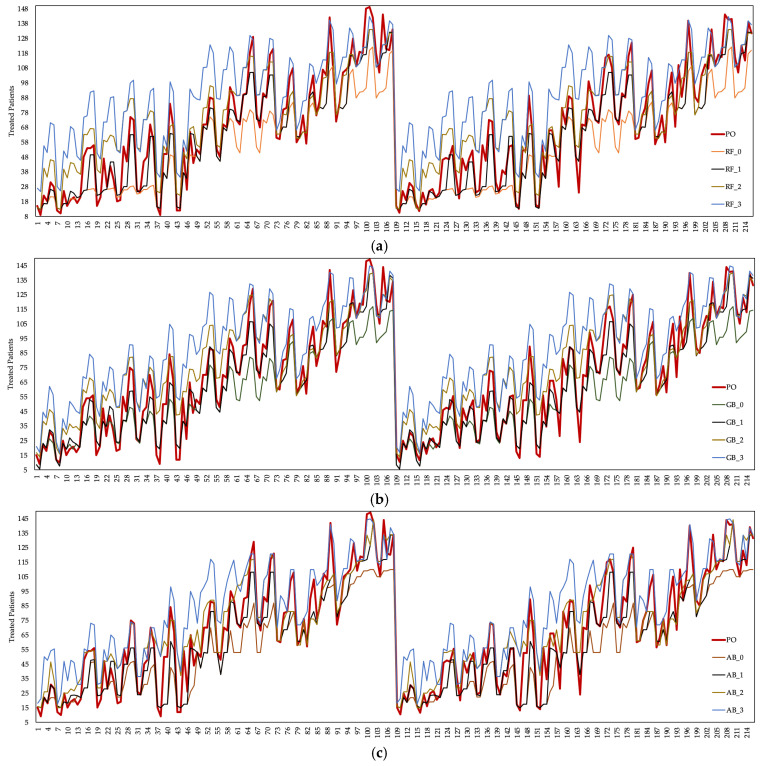
The estimated $p_{nt}$ based on the (**a**) RF algorithm ($\delta_{0.0}$ for $RF_{-0}$, $\delta_{0.1}$ for $RF_{-1}$, $\delta_{0.2}$ for $RF_{-2}$ for $RF_{-3}$), (**b**) GB algorithm ($\delta_{0.0}$ for $GB_{-0}$, $\delta_{0.1}$ for $GB_{-1}$, $\delta_{0.2}$ for $GB_{-2}$, $\delta_{0.3}$ for $GB_{-3}$) (**c**) AB algorithm ($\delta_{0.0}$ for $AB_{-0}$, $\delta_{0.1}$ for $AB_{-1}$, $\delta_{0.2}$ for $AB_{-2}$, $\delta_{0.3}$ for $AB_{-3}$).

The number of patients treated is generally defined as an output parameter in healthcare systems. In this study, $p_{nt}$ was considered an output parameter. Figure 5 shows the $p_{nt}$ based on the current situation and the estimation data obtained from the ML algorithms. There were fluctuations in the data obtained by running the DES model in the present case and the data obtained from the ML algorithms. Changes in the number of healthcare resources employed and the cost values of healthcare resources caused this situation to arise in the ED unit.

**Figure 6:**
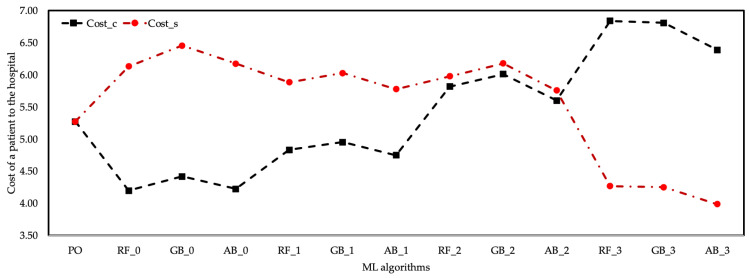
Cost of a patient to hospital according to $\delta_{i}$-based ML algorithms.

Among the prediction data of three different algorithms, the values of the AB algorithm contained values closer to the real data. It was observed that approximately 83 patients were treated, with a 0.218 MSE, 0.047 RMSE, 0.031 MAE in the training phase, and 2.740 MSE, 0.163 RMSE, 0.121MAE in the testing phase deviation of the average estimation data of the AB algorithm compared to the actual data. The prediction data of the RF and GB algorithms had an increase of 12% and 13%, respectively, compared to the actual data. The RF and GB algorithms had the highest $p_{nt}$ based on the $\delta_{0.3}$ coefficient. The AB algorithm is preferred over RF and GB algorithms since the statistical performance values of the AB algorithm are better than other algorithms. In general, it was observed that the average $p_{nt}$ increased as the cost coefficients of healthcare resources of all three algorithms increased. However, since the cost increase in the employment of healthcare resources was less than the income provided by the $p_{nt}$, it was understood that the cost of healthcare resources does not impose a burden on the hospital. Figure 6 shows the cost of a patient to the hospital according to the coefficient scenarios of the ML algorithms.

Despite the increase in the cost of healthcare resources according to the $\delta_{0.2}$ coefficient of the EU algorithm, the increase in the $p_{nt}$ was evaluated as the breakeven point of the cost of this scenario to the hospital. The cost of healthcare resources remained below the cost of the hospital with the increase in the number of patients treated according to the $\delta_{0.3}$ coefficient of all three ML algorithms. The $\delta_{0.3}$ coefficient of the AB algorithm provided the best cost performance of other algorithms.

### 3.4. The Estimated Waiting Times ($w_{t})$

**Figure 7:**
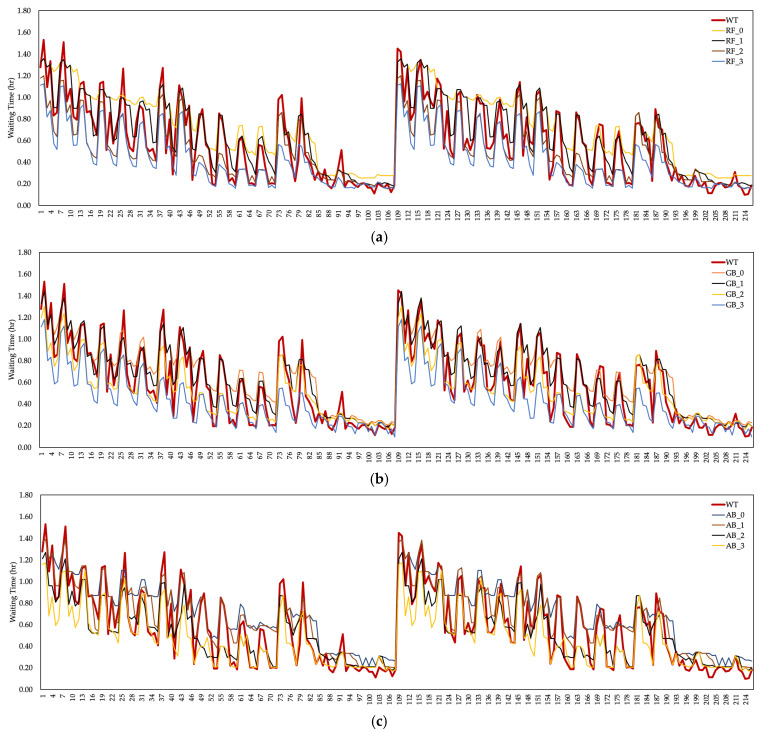
The value of $w_{t}$ based on the (**a**) RF algorithm ($\delta_{0.0}$ for $RF_{-0}$, $\delta_{0.1}$ for $RF_{-1}$, $\delta_{0.2}$ for $RF_{-2}$ for $RF_{-3}$), (**b**) GB algorithm ($\delta_{0.0}$ for $GB_{-0}$, $\delta_{0.1}$ for $GB_{-1}$, $\delta_{0.2}$ for $GB_{-2}$, $\delta_{0.3}$ for $GB_{-3}$) (**c**) AB algorithm ($\delta_{0.0}$ for $AB_{-0}$, $\delta_{0.1}$ for $AB_{-1}$, $\delta_{0.2}$ for $AB_{-2}$, $\delta_{0.3}$ for $AB_{-3}$).

Patient waiting times ($w_{t})$ resulting from overcrowding in an ED unit were considered one of the output parameters of this study. Although there are many reasons for $w_{t}$, the performance of healthcare resources or the number of employments had a significant impact. The estimation values of the ML algorithms are shown in Figure 7 to calculate the estimated data of the patient $w_{t}$ and the $p_{nt}$ by measuring the cost–performance relationship in the ED unit. According to the ML algorithms based on the $\delta_{0.2}$ coefficient, a significant decrease was observed in the $w_{t}$. The lowest value of $w_{t}$ was provided by the GB algorithm based on the $\delta_{0.3}$ coefficient. The maximum $w_{t}$ value was realized by the RF algorithm based on the $\delta_{0.0}$ coefficient. Fluctuations in the value of $w_{t}$ also affect the cost to the hospital.

**Figure 8:**
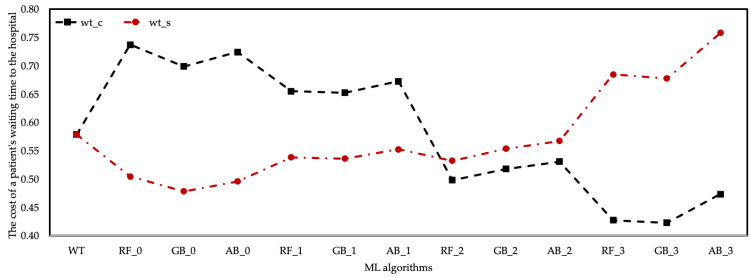
The cost to the hospital of the average $w_{t}$ is based on the $\delta_{i}$ coefficient.

Changes in $w_{t}$ according to healthcare resource cost coefficients are shown in Figure 8. There was a positive trend in the prediction $w_{t}$ data of ML algorithms based on the coefficients of $\delta_{0.0}$ and $\delta_{0.1}$. The same situation was observed in the estimation data of the RF algorithm based on the $\delta_{0.2}$ coefficient. $w_{t}$ are generally considered as costs. In this case, the cost of algorithms with high $w_{t}$ at the hospital should be evaluated. However, there was a decrease in $w_{t}$ based on the $\delta_{0.2}$ and $\delta_{0.3}$ coefficients, meaning the cost of the waiting period at the hospital increased.

It turns out that there was an inverse relationship between $w_{t}$, unlike the cost of patient treatment to the hospital, depending on the coefficient scenarios. In other words, the increase in the cost of the employed healthcare resources to the hospital and the decrease in the $w_{t}$ made it more costly to the hospital in total.

## 4. Discussions

This study presents that DES and ML algorithms can be used under a certain scenario to determine the $p_{nt}$ and $w_{t}$ from the dataset of hospitalized patients in ED. It has been demonstrated by this study that DES and ML algorithms can be very useful for calculating the estimated data of the generated scenarios and output variables. The DES model was created for the ED model of a hospital and the effects of the number and types of healthcare resources employed in the ED on $p_{nt}$ and $w_{t}$, as well as the $p_{nt}$ and $w_{t}$ values with the DES model were calculated. In the DES model, the data of patient arrival rates, the time of check-in and check-out, triage, examination by the nurses, and examination by the physicians were entered as distribution. Since the patients’ arrival to the ED unit of a hospital has different patient types and the duration of their appearance, the values of these parameters should be considered according to a distribution, not an integer or a certain ratio/time (such as a patient arrives every 10 min, treatment times 15 min, etc.). The DES model and ML algorithms were integrated since it is impossible to obtain $p_{nt}$ and $w_{t}$ estimation data in the DES model due to changes in healthcare resource costs.

Based on the results, it was observed that ML algorithms, RF, GB, and AB, with different properties, gave satisfactory results for the cost calculations of healthcare resources proposed in estimating, $p_{nt}$ and $w_{t}$. The results of the AB model, which offers the highest accuracy and the least margin of error, and other models were shared. Knowing that ML algorithms play an essential role in training data and memory footprints in obtaining statistical results, test and training datasets were run on models with 85–15%.

The results of this research on the $p_{nt}$ and $w_{t}$ prediction outputs of classification accuracy have proven that the AB algorithm is the best choice for a classification accuracy above 98%. The classification accuracy of other algorithms was calculated as over 96% (RF with 97.03%, and GB with 96.72). Although RF and GB achieved slightly lower classification accuracies than the AB algorithm, the accuracy rates of the prediction data of both were high. Average accuracy rates of RF, GB, and AB algorithms were calculated as 88.25%, 89.07%, and 89.70%, respectively. After all, what is common to all used classifiers is their high accuracy and low error rate. For increased accuracy and low error rates, the data used in the DES model must be correct, and the DES model needs to run correctly.

**Figure 9:**
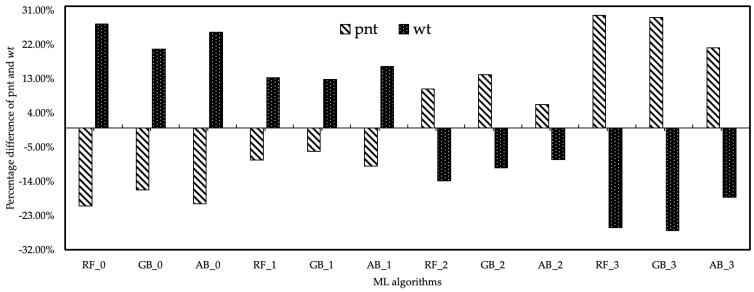
The fluctuation in the forecast data of ML algorithms based on the cost coefficient.

The cost analysis relationship between the output values of healthcare resource costs and the data obtained from the DES model and ML algorithms was also examined. According to the cost analysis of the results of ML algorithms based on the $\delta_{i}$ coefficient, it was determined that $p_{nt}$ increased with the increase in the healthcare resource cost coefficient for $p_{nt}$. Since the income provided by the $p_{nt}$ value was more than the healthcare resource cost, all scenarios created by the $\delta_{i}$ coefficient can be preferred. However, this does not apply to $w_{t}$. The $w_{t}$ estimation results of ML algorithms based on the $\delta_{i}$ coefficient differed. While the costs of $w_{t}$ values to the hospital were low in all ML algorithms with $\delta_{0.0}$ and $\delta_{0.1}$ coefficients, the cost of the hospital was increased by the $w_{t}$ values provided by ML algorithms based on $\delta_{0.2}$ and $\delta_{0.3}$ coefficients. However, the level of $w_{t}$ values of ML algorithms based on this coefficient was lower than the current $w_{t}$ level. As a result, fluctuations in the number of healthcare resources and cost coefficient in the cost analysis had a positive effect on the $p_{nt}$ value, while the oscillations had a negative effect on the $w_{t}$ value. The estimated data of $p_{nt}$ and $w_{t}$ were expressed as % of the results of the scenarios derived from the change in the cost coefficients of the healthcare resources. The developed method aimed to maximize the $p_{nt}$ number and minimize the $w_{t}$ amount among the findings of this study. Maximizing the $p_{nt}$ value and based on the data in the current system, the RF_3 algorithm provided 29.64% improvement. The GB_2 algorithm provided 26.89% improvement for $w_{t}$, and the minimum value of $w_{t}$ was obtained with this algorithm. Figure 9 expresses the percentage differences of fluctuation in the estimation data of ML algorithms based on the $\delta_{i}$ of healthcare resources.

In the present research, the average $w_{t}$ of a patient until the completion of all processes related to treatment/examination was calculated as 26.89% based on the GB algorithm. One study showed that with the integration of the DES and ML algorithms (Artificial Neural Network and Genetic Algorithm), the $w_{t}$ for the triage process approached the minimum, and the $w_{t}$ for a patient’s screening process was reduced from 158 min to 97 min (improvement of 62%). However, $w_{t}$ for other processes, such as doctor or nurse exams and check-in and check-out procedures, were not included in this study [32]. Another study used ML methods to estimate $w_{t}$ for patients in two emergency departments using data from more than 250 patients, resulting in a 24.85% improvement in $w_{t}$ based on the first scenario [30]. Lin et al. reduced the $w_{t}$ from 75.01 min to 68.39 min in patients with pupillary dilation and from 47.26 min to 44.54 min in patients without pupil dilation using RF and gradient boosting machine (GBM) algorithms [48]. Pak et al. reduced the number of patients with $w_{t}$ greater than 30 min by more than 42% with the predictive data using the quantile regression ML model [49]. A study using Logistic Regression, Extreme Gradient Boosting, Natural Gradient Boosting, SVM, and Decision Tree ML models reduced LOS by 12.3 min ($w_{t}$ reduction was achieved indirectly) from a general hospital’s emergency department in South Korea [50]. In terms of estimating $p_{nt}$, ML models are often used to estimate the number of patients for a disease type. Researchers mostly use DES models for $p_{nt}$ estimation. Alkhamis et al. developed the DES model and provided a 28% increase in the $p_{nt}$ [4]. Another study showed a 12% increase in $p_{nt}$ for high-volume colonoscopy screening using the DES technique under different scenarios [51]. Our study resulted in a 29.64% increase in $p_{nt}$ with the number of available healthcare resources and minimum cost.

On the whole, a method has been developed by applying the resource–cost–performance relationship, which is widely discussed in the healthcare system. In this study, the compatibility of ML algorithms with the DES model has been demonstrated by a numerical case study. In the proposed method, the cost-efficiency analysis process will inevitably provide detailed and tangible results in healthcare management, and this study will present cost recommendations for future periods.

## 5. Conclusions

Estimating unpredictable situations such as $w_{t}$, disease type, and patient arrival times in healthcare is closely related to ML algorithms. In ML algorithms, a certain output parameter estimation can be obtained when a data set is trained and tested with an appropriate ML algorithm. This kind of method makes a vital contribution to preventing the cases created by uncertain situations, especially by the healthcare experts. A second method that helps to predict uncertain situations is DES models designed in computer environment. In the healthcare system, which has a complex and dynamic structure, DES models provide great convenience to its users. This study delivered the integration of ML and DES models, and with these two models, accurate and sharp prediction data were obtained in advance of uncertain situations in the healthcare system in a fast and inexpensive way.

The main contribution of this study is the presentation of an important approach using ML and DES models to predict $p_{nt}$ and $w_{t}$ alongside traditional methods. This study represents the development of prediction models that use RF, GB, and AB machine learning algorithms to predict $p_{nt}$ and $w_{t}$ in light of the results obtained in the DES model using the dataset of patients arriving at the ED and compares the model performances. In addition, the effect of healthcare resource costs on $p_{nt}$ and $w_{t}$ and the cost to the hospital was analyzed with this compatible integration. This proposed approach provides an early estimation of the results obtained in determining the number of healthcare resources and the changes to be made in resource cost policies.

## Figures and Tables

**Table 1:** Studies related to the field of health management used by the DES model–ML algorithm.

Reference	Unit	ML Algorithms	The Purpose(s) of the Problem
[30]	ED	Naïve Bayes, Bayesian Networks, classification Trees	Examining patient satisfaction, waiting time estimation,
[31]	Hospitals, RCU	KNN, neural network, Decision Tree, Random Forest, Support Vector Machine	Improving health referrals processing
[32]	ED	Artificial Neural Network algorithm, Genetic Algorithm	Minimizing patients’ waiting time, the percentage of units’ engagement to enhance the ED efficiency
[33]	General *	XGBoost, Logistic Regression	Discovering optimal thresholds to effectively triage COVID-19 patients, minimizing death rates while preserving health system capacity.
[34]	ECC	Generalized Linear Model, Multivariate Adaptive Regression Splines, Random Forest, Support Vector Machine, Decision Tree	Analyzing critical crew capacity
[35]	HCU	K-Means Algorithm	To design health pathways and evaluate the return on investment of implementation
[36]	General *	Neural Network,	Development of healthcare information systems
This research	ED	Random Forest, Gradient Boosting, AdaBoost	Estimation of $p_{nt}$$\;\mathrm{and}\;w_{t}$, analysis of healthcare resources cost

Abbreviations: ECC, emergency coordination center; HCU, Hip fracture care unit; KNN, K-Nearest neighbor algorithm; RCU, the referral creation unit, * General issues related to the health system.

**Table 2:** The statistical characteristics of the input and output variables.

Features	$p_{n}$	$n_{n}$	$c_{n}$	$b_{n}$	$t_{n}$	$p_{nt}$	$w_{t}$
Status	Input	Input	Input	Input	Input	Output	Output
Data Type	Numeric *	Numeric *	Binary **	Numeric *	Binary **	Numeric *	Numeric
Mean	2.000	2.000	1.500	2.000	1.500	68.570	0.579
SE Mean	0.056	0.056	0.034	0.056	0.034	2.5500	0.024
StDev	0.818	0.818	0.501	0.818	0.501	37.550	0.358
Variance	0.670	0.670	0.251	0.670	0.251	1409.9	0.128
CoefVar	40.92	40.92	33.41	40.92	33.41	54.760	61.78
Minimum	1.000	1.000	1.000	1.000	1.000	9.0000	0.098
Median	2.000	2.000	1.500	2.000	1.500	68.000	0.539
Maximum	3.000	3.000	2.000	3.000	2.000	149.00	1.530
Skewness	0.000	0.000	0.000	0.000	0.000	0.2100	0.510
Kurtosis	−1.510	−1.510	−2.020	−1.510	−2.020	−0.950	−0.720

* Integer, ** binary: [1,2], and integer.

**Table 3:** Healthcare resources employed in the ED unit and their characteristics.

Staff	Gender	Quantity	Type	Responsible Process	Process Distribution
Physicians	Female/Male	3	Human-based	diagnosis/examination/treatment	Uniform (10.0, 30.0, 0.0)
Nurses	Female/Male	3	Human-based	assisting the doctor, to follow the patient, provide control, triage process, perform additional operations *	Triangular (3.0, 15.0, 5.0)
Clerks	Female/Male	2	Human-based	check-in/check-out	Uniform (3.0, 5.0, 0.0)
Beds or Exam Rooms	-	5	Location-based	the area reserved for the patient during the treatment or examination process	Triangular (3.0, 15.0, 5.0) ** Uniform (10.0, 30.0, 0.0)
Triage areas and equipment	-	2	Location-based	the area where the patient’s first health check is provided, and the values are measured	Triangular (3.0, 15.0, 5.0)
Waiting Room	-	1 (Limit: 50 seats)	Location-based	the area where patients wait until health resources become available	Triangular (0.0, 0.18, 1.68) ***

* Injection, serum, medicine support, providing extra information, etc. ** the duration of the procedures performed by the nurses. *** hr.

**Table 4:** $\alpha_{i}$ values that affect the cost of the unit healthcare resource to the ED unit.

Parameters	$p_{n}$	$n_{n}$	$c_{n}$	$b_{n}$	$t_{n}$	%	Cumulative %
$\delta_{0.0}$	1.00	0.80	0.60	0.50	0.40	0.00	1.00
$\delta_{0.1}$	1.20	0.96	0.72	0.60	0.48	0.20	1.20
$\delta_{0.2}$	1.56	1.25	0.94	0.78	0.62	0.30	1.30
$\delta_{0.3}$	2.34	1.87	1.40	1.17	0.94	0.50	1.50
Sum	6.10	4.88	3.66	3.05	2.44	1.00	5.00

**Table 5:** Correlation values between variables according to the $\delta_{i}$ values.

$\delta_{i}$ Values	**Feature 1**	Feature 2	Feature 3	$R_{p_{nt}}\;$	$R_{w_{t}}\;$
$\delta_{0.0}$	$p_{n}\;$	$p_{nt}$	$w_{t}$	0.844	−0.726
	$n_{n}\;$	$p_{nt}$	$w_{t}$	0.372	−0.540
	$b_{n}\;$	$p_{nt}$	$w_{t}$	0.328	−0.293
	$c_{n}\;$	$p_{nt}$	$w_{t}$	−0.015	−0.246
	$t_{n}\;$	$p_{nt}$	$w_{t}$	−0.211	0.232
$\delta_{0.1}\;$	$p_{n}\;$	$p_{nt}$	$w_{t}$	0.777	−0.682
	$n_{n}\;$	$p_{nt}$	$w_{t}$	0.456	−0.538
	$b_{n}\;$	$p_{nt}$	$w_{t}$	0.381	−0.397
	$c_{n}\;$	$p_{nt}$	$w_{t}$	−0.218	−0.247
	$t_{n}\;$	$p_{nt}$	$w_{t}$	−0.210	0.227
$\delta_{0.2}\;$	$p_{n}\;$	$p_{nt}$	$w_{t}$	0.722	−0.622
	$n_{n}\;$	$p_{nt}$	$w_{t}$	0.559	−0.584
	$b_{n}\;$	$p_{nt}$	$w_{t}$	0.299	−0.381
	$c_{n}\;$	$p_{nt}$	$w_{t}$	−0.261	−0.225
	$t_{n}\;$		$w_{t}$	−0.201	0.201
$\delta_{0.3}\;$	$p_{n}\;$	$p_{nt}$	$w_{t}$	0.774	−0.627
	$n_{n}\;$	$p_{nt}$	$w_{t}$	0.424	−0.351
	$b_{n}\;$	$p_{nt}$	$w_{t}$	0.367	−0.506
	$c_{n}\;$	$p_{nt}$	$w_{t}$	−0.275	−0.281
	$t_{n}\;$	$p_{nt}$	$w_{t}$	−0.201	0.218

**Table 6:** Values of measurement performances of ML algorithms for $p_{nt}$ and $w_{t}$.

Outputs	Algorithm	Training Dataset				Testing Dataset			
		MSE	RMSE	MAE	R^2^	MSE	RMSE	MAE	R^2^
$p_{nt}$	RF	0.4022 *	0.0634 *	0.0463 *	0.9703	0.7830 *	0.0885 *	0.0699 *	0.9539
	GB	0.4433 *	0.0666 *	0.0511 *	0.9672	0.8457 *	0.0920 *	0.0721 *	0.9502
	AB	0.2185 *	0.0467 *	0.0307 *	0.9838	0.3107 *	0.0557 *	0.0385 *	0.9817
$w_{t}$	RF	0.0089	0.0943	0.0704	0.9275	0.0096	0.0981	0.0732	0.9216
	GB	0.0098	0.0988	0.0746	0.9205	0.0098	0.0988	0.0746	0.9205
	AB	0.0062	0.0789	0.0566	0.9492	0.0062	0.0789	0.0566	0.9492

Consider MSE, RMSE, and ME as %, * per 100 patients.

**Table 7:** Values of measurement performances of ML algorithms for $p_{nt}$ and $w_{t}$ based on $\delta_{i}$.

Outputs	$\delta_{i}\;\mathrm{Values}$	Algorithm	Training Dataset				Testing Dataset			
			MSE	RMSE	MAE	R^2^	MSE	RMSE	MAE	R^2^
$p_{nt}$	$\delta_{0.0}$	RF	0.4194	0.0648	0.0466	0.9690	3.3397	0.1827	0.1395	0.7620
		GB	0.4433	0.0666	0.0511	0.9672	2.8690	0.1694	0.1315	0.7956
		AB	0.2185	0.0467	0.0307	0.9838	3.7999	0.1949	0.1491	0.7292
	$\delta_{0.1}$	RF	0.4086	0.0639	0.0446	0.9709	1.4427	0.1201	0.0891	0.8972
		GB	0.4390	0.0663	0.0498	0.9687	0.9857	0.0993	0.0759	0.9298
		AB	0.2207	0.0470	0.0306	0.9843	1.6923	0.1301	0.0915	0.8794
	$\delta_{0.2}$	RF	0.4273	0.0654	0.0466	0.9684	2.9333	0.1713	0.1387	0.7910
		GB	0.4433	0.0666	0.0511	0.9672	2.4027	0.1550	0.1285	0.8288
		AB	0.2185	0.0467	0.0307	0.9838	1.8117	0.1346	0.0945	0.8709
	$\delta_{0.3}$	RF	0.4127	0.0642	0.0460	0.9706	6.2546	0.2501	0.2116	0.5543
		GB	0.4390	0.0663	0.0498	0.9687	5.7007	0.2388	0.2068	0.5938
		AB	0.2160	0.0465	0.0307	0.9846	3.6574	0.1912	0.1506	0.7394
$w_{t}$		RF	0.0089	0.0944	0.0690	0.9300	0.0150	0.1226	0.0917	0.8818
		GB	0.0094	0.0972	0.0738	0.9258	0.0150	0.1225	0.0929	0.8820
		AB	0.0062	0.0787	0.0571	0.9514	0.0193	0.1390	0.0996	0.8482
	$\delta_{0.1}$	RF	0.0086	0.0930	0.0692	0.9320	0.0163	0.1276	0.0948	0.8720
		GB	0.0094	0.0972	0.0738	0.9258	0.0150	0.1225	0.0929	0.8821
		AB	0.0061	0.0784	0.0578	0.9517	0.0197	0.1403	0.1004	0.8454
	$\delta_{0.2}$	RF	0.0085	0.0923	0.0666	0.9331	0.0162	0.1272	0.0939	0.8728
		GB	0.0094	0.0972	0.0738	0.9258	0.0150	0.1225	0.0929	0.8819
		AB	0.0062	0.0787	0.0571	0.9514	0.0193	0.1390	0.0996	0.8482
	$\delta_{0.3}$	RF	0.0083	0.0914	0.0677	0.9344	0.0151	0.1231	0.0928	0.8810
		GB	0.0094	0.0972	0.0738	0.9258	0.0150	0.1225	0.0929	0.8818
		AB	0.0062	0.0787	0.0571	0.9514	0.0193	0.1390	0.0996	0.8482

## Data Availability

Not applicable.
